# A Traumatic Loss of the Calcaneus and Heel Pad From a Lawnmower Injury in a Pediatric Patient: A Case Report

**DOI:** 10.7759/cureus.100579

**Published:** 2026-01-01

**Authors:** Quintin Norris, John D Murphy, William R Judson, Anthony Florschutz

**Affiliations:** 1 Orthopedic Surgery, Nova Southeastern University Dr. Kiran C. Patel College of Osteopathic Medicine, Clearwater, USA; 2 Orthopedic Surgery, Largo Medical Center, Largo, USA

**Keywords:** anterolateral thigh skin graft, degloving injury, growth plate preservation, heel pad avulsion, hindfoot reconstruction, lawnmower trauma, limb salvage, pediatric calcaneal injury, pediatric foot trauma, staged reconstruction

## Abstract

Traumatic calcaneal injuries with combined soft tissue loss are extremely rare in pediatric patients, posing significant reconstructive challenges due to contamination and the need to preserve growth potential. We report a case of a 10-year-old female who sustained a lawnmower-induced degloving injury with partial calcaneal loss, plantar heel pad avulsion, and posterior calf soft tissue damage. Management included emergent debridement followed by staged reconstruction aimed at preserving the distal calcaneal growth plate. At 11 months post-injury, the patient achieved complete wound closure, independent ambulation, a plantigrade foot, and functional ankle motion without infection or growth disturbances.

This case is unique due to the combination of bony and soft tissue loss, high contamination, and pediatric growth plate considerations. It demonstrates that even severe pediatric hindfoot injuries can be managed successfully with tailored, multidisciplinary reconstruction, achieving limb salvage while preserving function and growth potential.

## Introduction

The calcaneus is the largest and most robust bone of the foot, closely associated with the heel pad and surrounding soft tissues that are critical for maintaining hindfoot integrity [1]. Traumatic calcaneal injuries in children are exceedingly rare, accounting for approximately 0.005%-0.41% of all pediatric fractures [2]. Complete or near-complete loss of the calcaneus in pediatric patients is exceptionally uncommon, with only a handful of cases of complete calcaneal loss and reconstruction reported in the literature [3,4]. Most published reports of total or near-total calcaneal reconstruction involve adult patients, in whom management typically includes amputation, structural bone grafting, or prosthetic reconstruction to restore hindfoot alignment and function [5,6]. In contrast, calcanectomy in pediatric patients is most performed for tumor‑related pathology rather than traumatic injury, reflecting both the distinct reconstructive considerations of the skeletally immature hindfoot and the rarity of traumatic calcaneal loss in children [7,8].

The scarcity of available data provides limited guidance for the management of traumatic calcaneal loss in skeletally immature patients. Pediatric reconstruction presents unique challenges, including the presence of an open distal calcaneal physis, which limits options for extensive bony reconstruction without compromising growth potential [9]. Additionally, prosthetic use in children is complicated by ongoing growth, limb‐length changes, and evolving skeletal morphology [10]. When heel‐pad loss is present, the complexity of reconstruction is substantially increased, necessitating concurrent planning and execution by orthopedic and plastic surgical teams to restore bone and soft‑tissue form and function, optimize weight‐bearing capacity, and preserve long‐term mobility [11].

This report aims to describe the multidisciplinary reconstructive management and outcome of a 10-year-old female with combined traumatic loss of the calcaneus and heel pad following a lawnmower injury. This case highlights the surgical decision-making process, reconstructive approach, and postoperative rehabilitation considerations that guided preservation of limb function and growth potential.

## Case presentation

**Figure 1:**
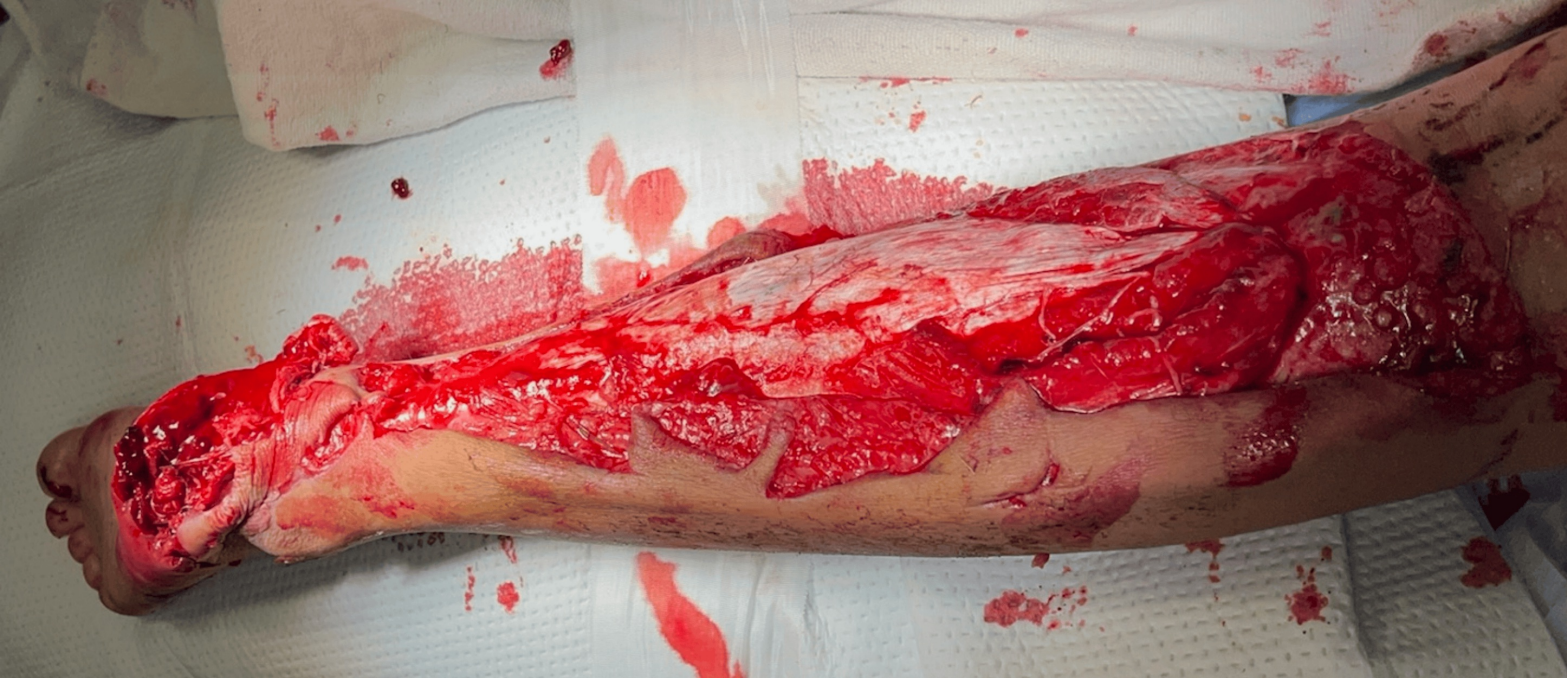
Preoperative clinical photograph demonstrating an extensive degloving injury extending from the posteromedial heel to the distal thigh (distal foot positioned to the left). The plantar heel pad is completely separated from the underlying fascia, with soft tissue gaps across the plantar surface.

A 10-year-old female sustained a domestic lawnmower injury, causing extensive soft tissue degloving below the right knee. On arrival, she was alert and hemodynamically stable. Examination revealed a large posterior distal leg and ankle wound with multifocal soft tissue loss extending below the knee (Figure 1). Distal pulses were palpable, capillary refill was normal, and motor and sensory function were intact.

**Figure 2:**
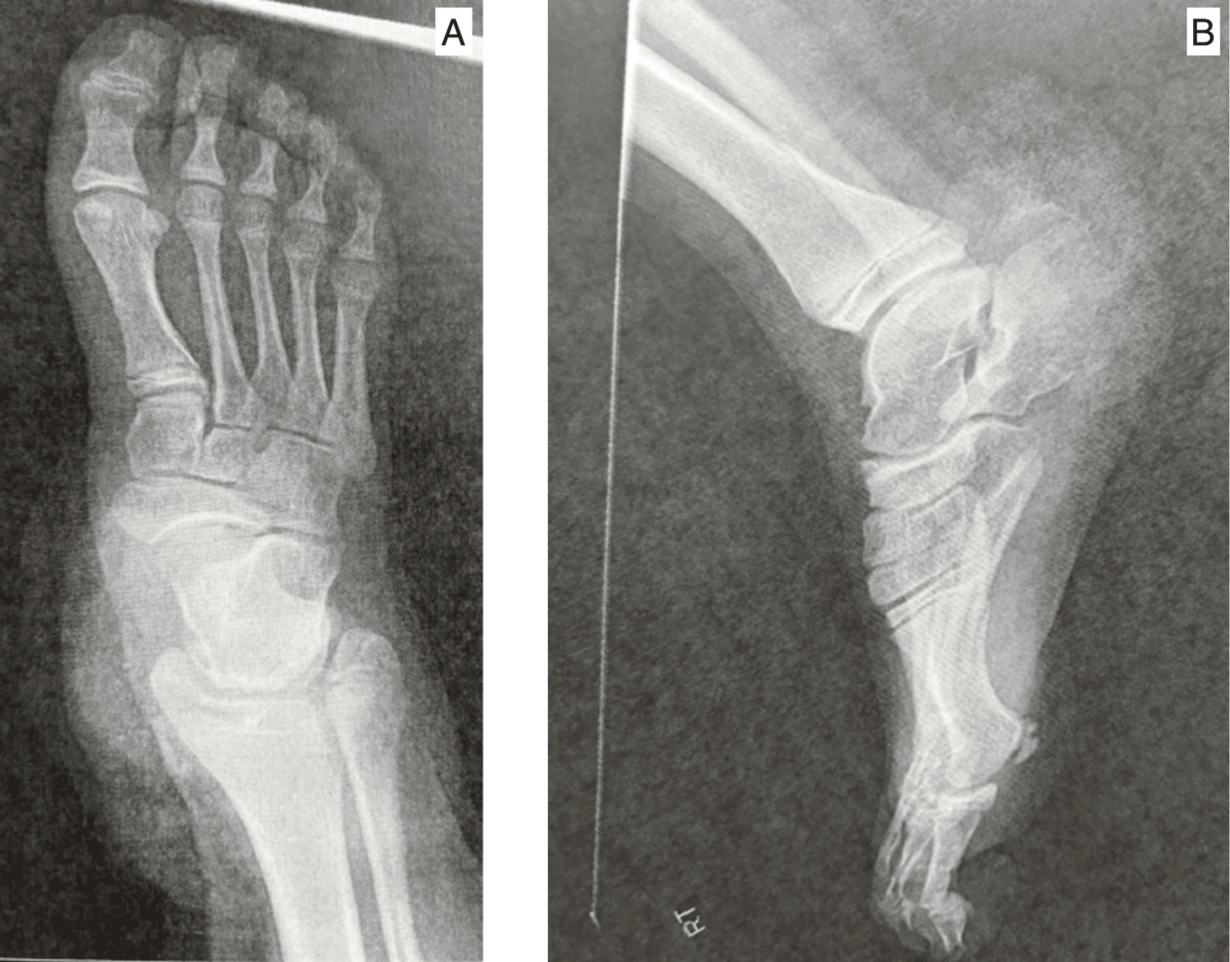
(A) Anteroposterior and (B) lateral radiographs of the right foot showing calcaneal tuberosity amputation and associated posterior soft tissue defect.

Radiographs demonstrated amputation of the calcaneal tuberosity extending into the posterior subtalar joint, without additional fractures (Figure 2). Empiric intravenous antibiotics were initiated, and emergent irrigation and debridement were performed. Intraoperative findings included an open wound >50 cm², Achilles tendon avulsion, loss of one-third of the calcaneus, organic debris, and transected medial and lateral gastrocnemius with ~25% muscle belly loss.

**Figure 3:**
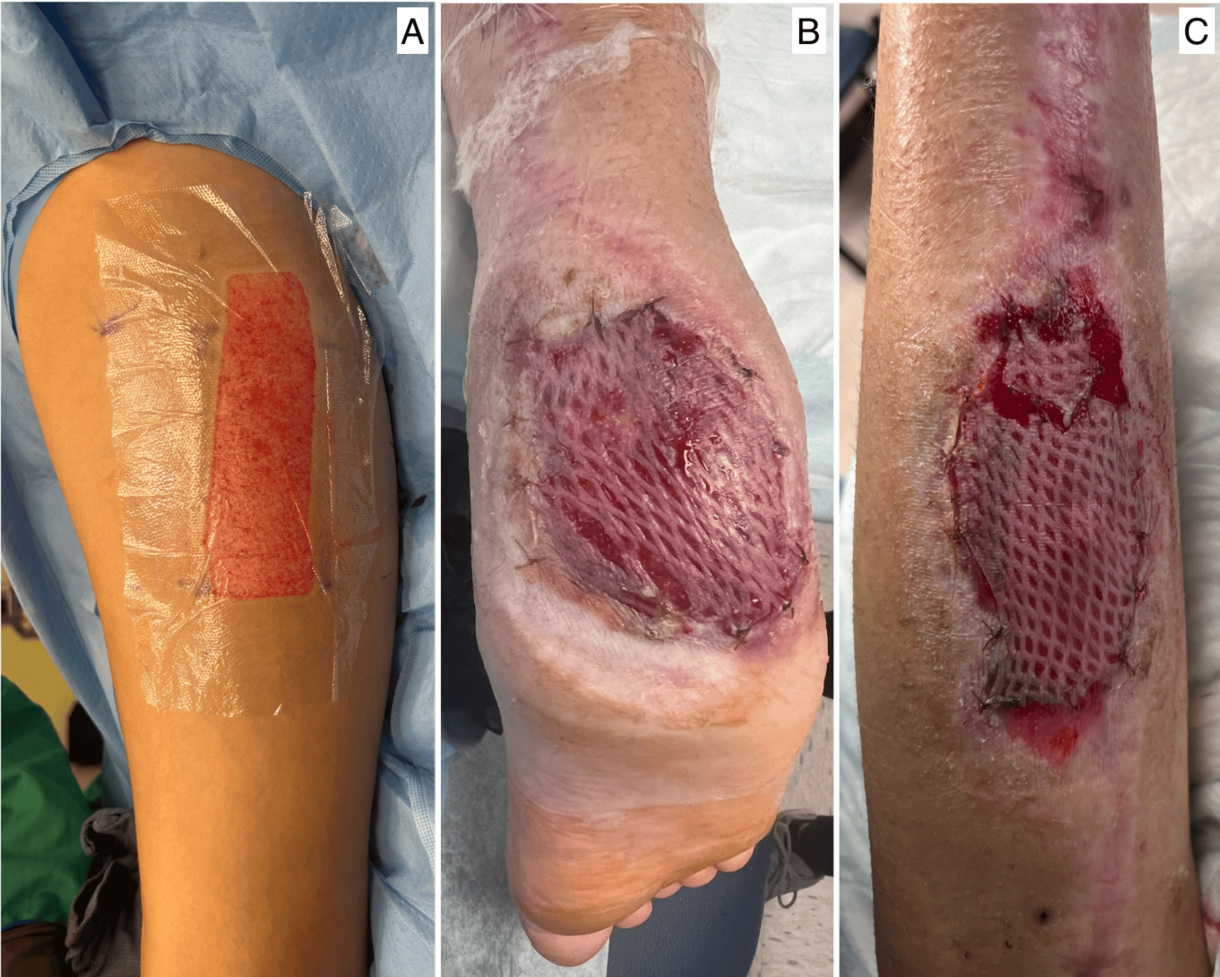
Postoperative photographs showing (A) harvested anterolateral thigh flap (ALT) 69 days post-injury, (B) ALT flap placement over mid-calf wound, and (C) ALT flap placement over plantar heel at 75 days post-injury.

A biologic adjunct, AMNIOFIX® (MiMedx Group, Inc.), was used to promote soft-tissue healing and support the underlying tendon and fascial repair. Serial wound management included staged debridement and negative-pressure therapy (Table 1). By day 53, robust granulation tissue covered the defect. Definitive coverage was achieved with a thigh-based fasciocutaneous flap inset onto the heel, supplemented with skin grafting and protective dressings (Figure 3).

**Table 1:** Summary of serial debridement, VAC therapy, and AMNIOFIX® applications VAC: vacuum-assisted closure; CAM: controlled ankle-motion boot; ALT: anterolateral thigh flap AMNIOFIX®, MiMedx Group, Inc.

Postoperative day	Intervention / procedure	Notes / outcome
0	Initial irrigation and debridement; partial closure; VAC placement	Calcaneal fragment tagged; CAM boot applied
2	Repeat irrigation; VAC exchange	Minor dusky areas; healthy granulation tissue
5	Debridement; Achilles & plantar fascia repair; AMNIOFIX® application; dual VAC placement	Two suture anchors; Krackow technique used
12–33	Debridement; VAC exchange; AMNIOFIX® application	Progressive granulation tissue formation
53	Debridement; VAC exchange; AMNIOFIX® application	Complete granulation achieved; ready for definitive coverage
54+	ALT flap and skin graft; ReCell + VAC applied	Heel defect covered; flap inset successfully

Outpatient follow-up included routine wound checks and dressing changes. At four months, a custom orthotic was provided for ambulation. The final procedure, performed 124 days post-injury, involved suture removal and minor local revision. At 11 months, the wound was fully closed, and independent ambulation was achieved. A mild secondary deformity was evident, attributable to partial loss of heel height. Without orthotic support, ambulation demonstrated a mildly altered gait secondary to leg-length discrepancy; with insoles, a plantigrade, functional gait was maintained. Ankle range of motion was preserved for age, allowing weight bearing and functional mobility. Radiographs confirmed stable bony reconstruction without evidence of growth plate disturbance, infection, or complications. Annual follow-up is planned to monitor long-term outcomes, including gait, limb length, and degenerative changes.

## Discussion

Severe pediatric calcaneal injuries involving both osseous and soft-tissue components are exceptionally challenging because of contamination, irregular tissue margins, and the need to preserve growth potential [9,12]. In this case, a 10-year-old female sustained a lawnmower-induced degloving injury with partial calcaneal loss, plantar heel pad avulsion, and posterior calf soft tissue damage, which was managed through a staged, multidisciplinary approach to optimize limb function and growth.

Limb salvage was prioritized over partial calcanectomy or amputation because of the preserved distal calcaneal fragment, intact neurovascular status, and potential for normal weight-bearing and gait. Although more radical approaches could have removed necrotic tissue definitively, they are associated with chronic pain, prosthetic dependency, and challenges in accommodating skeletal growth in pediatric patients [13]. Early collaboration between orthopedic and plastic surgery teams facilitated meticulous soft tissue management, staged debridement, and strategic use of AMNIOFIX® and vacuum-assisted closure (VAC) therapy to optimize tissue viability while protecting the distal calcaneal physis [11,14].

Prior pediatric cases have demonstrated reconstruction using pedicled fibular growth plate flaps, isolated heel pad repair, or anterolateral thigh flap (ALT) free flaps, such as a 5-year-old with complete calcaneal loss reconstructed via a fibular growth plate flap, an 8-year-old with isolated heel pad avulsion, and a 4-year-old who underwent ALT free flap reconstruction after a lawnmower injury [12,15,16]. In this patient, extensive posterior calf soft tissue loss precluded fibular growth plate flap use, and combined calcaneal and heel pad loss made isolated soft tissue coverage insufficient. These factors highlight the need for a tailored, staged reconstructive strategy.

The preservation of the distal calcaneal physis was a critical consideration. Radiographs and functional follow-up at 11 months demonstrate an intact growth plate and stable alignment, with no evidence of limb length discrepancy or deformity. Ongoing annual follow-up is necessary to monitor for potential growth disturbances, calcaneal remodeling, or degenerative changes. Early weight-bearing with orthotic support and structured rehabilitation may mitigate gait adaptations and functional deficits [17,18].

This case illustrates that individualized, multidisciplinary planning can achieve limb salvage with functional ambulation, a plantigrade foot, and maintained growth potential, even in complex, contaminated pediatric hindfoot injuries. The approach described here provides a potential framework for managing similar injuries in skeletally immature patients, balancing limb preservation with long-term functional outcomes.

## Conclusions

This case demonstrates short-term limb salvage in a pediatric patient with traumatic heel degloving and partial calcaneal loss using staged debridement, ALT flap coverage, and orthotic-supported rehabilitation. Preliminary outcomes suggest preservation of the distal growth plate and age-appropriate ambulation, allowing return to daily activities with orthotic support. Clinicians managing similar injuries should consider early multidisciplinary collaboration, staged debridement and soft tissue coverage, and orthotic-supported rehabilitation to support bone preservation, growth potential, and functional recovery.
